# The Oxidative Paradox in Low Oxygen Stress in Plants

**DOI:** 10.3390/antiox10020332

**Published:** 2021-02-23

**Authors:** Chiara Pucciariello, Pierdomenico Perata

**Affiliations:** PlantLab, Institute of Life Sciences, Scuola Superiore Sant’Anna, 56127 Pisa, Italy; pierdomenico.perata@santannapisa.it

**Keywords:** anoxia, hypoxia, nitric oxide, NO, reactive oxygen species, ROS

## Abstract

Reactive oxygen species (ROS) are part of aerobic environments, and variations in the availability of oxygen (O_2_) in the environment can lead to altered ROS levels. In plants, the O_2_ sensing machinery guides the molecular response to low O_2_, regulating a subset of genes involved in metabolic adaptations to hypoxia, including proteins involved in ROS homeostasis and acclimation. In addition, nitric oxide (NO) participates in signaling events that modulate the low O_2_ stress response. In this review, we summarize recent findings that highlight the roles of ROS and NO under environmentally or developmentally defined low O_2_ conditions. We conclude that ROS and NO are emerging regulators during low O_2_ signalling and key molecules in plant adaptation to flooding conditions.

## 1. Introduction

A low oxygen (O_2_) availability characterized the atmosphere of Earth for most of its history [1]. Land plants evolved from algae around 500 million years ago [2], and the O_2_ content available today in the atmosphere is currently attributed to this event [3]. Like most aerobic organisms, plants harbour and use enzymes for O_2_-dependent energy metabolism, and the production of adenosine triphosphate (ATP) from glucose is higher when enough O_2_ is present.

Environmental conditions, such as excessive precipitation events, can lead to spatio-temporal limitation of O_2_ availability for roots or for the entire plant [4]. Under O_2_ shortage, plant functions are compromised. In order to adapt and survive low O_2_, plants sense the O_2_ level and adopt strategies that range from metabolic adjustments to morphological adaptations.

Under aerobic conditions (i.e., around 20% O_2_), several pathways, including those for energy production, give rise to reactive oxygen species (ROS) [5]. In this sense, the presence of ROS at the same time as low O_2_ availability is apparently contradictory. In spite of this, the presence and activity of ROS have been detected in plant systems as a consequence of O_2_ shortage and even under anoxia [6]. These observations recall the process occurring in mammalian cells, where ROS are present under hypoxia and are involved in the modulation of hypoxic signalling [7].

ROS are well known for their contrasting function as an adaptive signal aimed at a stress response and, at the same time, eventually leading to cell death when their production is not under homeostatic control [5]. This aspect of ROS is particularly challenging during O_2_ shortage, as it is difficult to distinguish between the plant’s adaptive and dysfunctional response.

Nitric oxide (NO) was, similarly to ROS, formerly considered to be harmful to cells [8] and now a key component of signal transduction networks [9]. In plants, NO is involved in the degradation of the transcriptional regulators that drives the activation of the core hypoxic genes [10]. In fact, NO availability negatively regulates the activation of the response to anaerobiosis driven by group VII ethylene responsive factors (ERF-VII) [10]. This function has not been experimentally proved for ROS in plants, but nor has it been ruled out [11].

Direct O_2_ sensing occurs through the plant cysteine oxidase (PCO) dependent oxidation of N-terminal Cys of ERF-VII proteins [12], ROS are known to participate in low O_2_ adaptive mechanisms by plants, such as during adventitious root (AR) emergence and aerenchyma formation.

NO production and turnover in mitochondria are also involved in the phytoglobin/NO pathway for the production of ATP when O_2_ is low, through the generation of an electrochemical gradient that involves nitrite conversion to NO [13]. Recently, the isolation of mitochondria from pea (*Pisum sativum*) and the treatment with nitrite under hypoxia, showed an increase in NO production that was linked to preserved mitochondria integrity, increased ATP synthesis and reduced ROS production [14].

In parallel to the activation of the anaerobic response in the presence of an environmental-dependent O_2_ shortage (waterlogging, flooding), plants also harbour hypoxic niches even when grown under normal O_2_ availability [15,16]. These hypoxic compartments are believed to be required to drive cellular proliferation and differentiation. O_2_ gradients are endogenously generated possibly as a consequence of the anatomy and/or the physiology of particular tissues, where the homeostatic state of hypoxia can be classified as “chronic” [17]. In this context, the role of ROS, if any in relation to hypoxia, has not been clarified [8].

ROS/NO are also involved in plant-microbe interactions. Recent results identified interfaces between plants and microbes as hypoxic microenvironments, such as necrotic areas, the gall tissue, and the symbiotic nodule structure [18]. The possible interaction between the plant-microbe derived hypoxic niches and the ROS/NO signature is currently not known. There are therefore still many open questions and the topic of low O_2_/oxidative burst paves the way for several working hypotheses.

## 2. ROS/NO Role in Hypoxia Sensing

In plants, the presence of O_2_ results in the instability of ERF-VII [19,20] with members of this transcription factor family, namely RAP2.12 and RAP2.2, playing a key role in activating the anaerobic response at the transcriptional level [21]. The stability of these transcription factors (TFs) is controlled by oxidation at their N-terminal Cys residue, a reaction catalysed by PCOs [12]. When O_2_ is available, after Met removal, the Cys located in N-terminal position is oxidized, due to the O_2_-dependent enzymatic reaction guided by PCOs, which is essential for subsequent protein arginylation. The arginylated protein is then recognised by an E3 ubiquitin-protein ligase (PRT6), which drives its ubiquitin-associated proteasome degradation.

The N-degron pathway for O_2_ sensing in plants resembles the hypoxia inducible transcription factors (HIFs) regulatory system in animals [22]. HIFs are heterodimers composed of an α subunit, post-transcriptionally regulated by O_2_ availability, and a β subunit, which is constitutively expressed [23]. When O_2_ is available, HIF-α is hydroxylated by O_2_ dependent prolyl hydroxylases (PHD) and the factor-inhibiting HIF (FIH) asparaginyl hydroxylase, and becomes a target for ubiquitin ligase complexes for the subsequent proteasome-dependent degradation [24,25,26,27,28]. Under O_2_ shortage, the HIF1 complex is reconstituted at the nucleus where it drives the expression of hypoxic genes [29].

In animals, ROS have long been considered to have a regulatory role under hypoxia, stabilising HIF1 [7]. Early experiments on mammalian cells with antioxidants and mitochondria chemical inhibitors (e.g., antimycin A) suggested that hypoxia results in the production of ROS by mitochondria with the involvement of complex I and III of the mitochondrial electron transport chain (mETC) [30,31]. Subsequent experiments using genetic approaches strengthened this hypothesis [32,33] and suggested that ROS signals could inhibit PHD and FIH, thus mediating HIF stabilisation [34,35]. A similar mechanism may be present in plants, operating on possible negative regulators of the ERF-VIIs thus contributing indirectly to their stabilization.

Very recently, live monitoring of cytosolic response in Arabidopsis leaves under hypoxia using a multiwell platform and fluorescent-based sensor proteins, highlighted in vivo dynamics of the cell physiological state [36]. Comparing hypoxia response with pharmacological inhibition of mETC, the authors identified impaired respiration as a key cause of several molecular changes under low O_2_ [36]. The chemical treatment applied to Arabidopsis leaves consisted of antimycin A, an inhibitor of mETC complex III, alone or combined with salicylhydroxamic acid (SHAM), which inhibits plant alternative oxidase (AOX). These treatments led to modifications in cytosolic sensors response similar to those of a hypoxia treatment, suggesting a common mechanism. Among the sensors, the state of oxidation of glutathione through the cyt-Grx-roGFP2 sensor strikingly increased at the onset of hypoxia, reaching a plateau that was long lasting. One of the possibilities is that this oxidation state of glutathione may be due to ROS increase and thus detoxification activity by glutathione pool [37].

The power of the multiplexing approach and the possibility to transfer the hypoxia-related mitochondrial signaling model to a natural context has been recently discussed [37], highlighting the interest in using further biosensors for candidates of signaling under hypoxia, such as the roGFP2-Orp1 fluorescent protein sensor to monitor hydrogen peroxide (H_2_O_2_) [38].

In parallel, the variation in the redox state of the cell was shown to promote redox-dependent post translational modification of Cys residues (Cys47 and Cys 243) on Arabidopsis ADH that influence the enzyme activity [39]. Recently, Arabidopsis ADH1 and ADH2 Cys47 were found to be S-sulfenylated, suggesting Cys47 to act as an H_2_O_2_-sensitive switch for ADH enzymatic activity [40]. Interestingly, the activity of the ADH enzyme was found to be dampened in *atrbohD*, *atrbohF* and *atrbohD1/F1* Arabidopsis mutants under hypoxia [41]. This indicates a level of post-transcriptional regulation of hypoxia-related enzymes that may be independent of their transcriptional regulation by ERF-VII and related to the cellular redox state.

NO is known to be involved in the plant’s O_2_ sensing. Using pharmacological and genetic tools, it has been demonstrated that, together with O_2_, NO is in fact responsible for the degradation of ERF-VII. Arabidopsis *nia1nia2noa1-2* mutants, impaired in the production of NO, show the transcription of anaerobic genes, and *nia1nia2* mutants display the stabilisation of the ERF-VII member hypoxia-responsive ERF2 HRE2 [10]. NO enhances ERF-VII instability acting presumably downstream of PCO activity. In fact, PCO enzymes do not require NO for their activity in vitro [12,42]. In yeast, the synthetic reporter for the O_2_ level dual-luciferase O_2_ reporter (DLOR), which is based on the ERF-VII/PCO4 system, was used together with the NO donor S-nitroso-*N*-acetyl-DL-penicillamine (SNAP) and NO scavenger 2–4-carboxyphenyl-4,4,5,5-tetramethylimidazoline-1-oxyl-3-oxide (cPTIO) [43]. SNAP and cPTIO did not affect the DLOR stability, suggesting that PCO4 does not require NO to be able to degrade proteins harbouring the N-degron.

It is still unknown whether NO plays a role in enzymatic or non-enzymatic oxidation of ERF-VIIs in plants and whether this mechanism is devoted to the exclusive modification of the Cys located at the N-terminal protein site. NO is able to convert Cys residues to S-nitrosothiols, and this process can involve O_2_ or its derivatives [44]. This could represent an additional mechanism that regulates the stability of ERF-VII proteins. In mammalian cells, proteins regulated by the Cys-branch of the N-degron pathway require NO before arginylation [44].

In Arabidopsis, early ethylene entrapment due to submergence increases transcription of the NO-scavenger non-symbiotic phytoglobin 1 (PGB1), thus reducing the amount of NO availability and promoting ERF-VII stability [45]. This event occurs prior to severe hypoxia and, acting as a priming event, enhances plant tolerance to the forthcoming stress. It would thus be interesting to test whether and how the PGB1 mechanism operates in the absence of ethylene entrapment, i.e., in developmental hypoxic niches.

At the onset of anoxia, a burst of ROS is produced in Arabidopsis, likely as a consequence of membrane NADPH-oxidase activity [46] and mETC imbalance [47]. This imbalance activates downstream mitogen-activated protein kinases (MAPKs) [47]. Following the activation of ROS pathways, heat shock factors (HSFs) and small heat shock proteins (HSPs) are transcribed [48]. HSFs and HSPs likely help to protect cells under anoxia, which therefore overlaps to some extent with the response to heat stress [49].

Mitochondria-dependent signaling is crucial to low O_2_ stress response in plants [37]. Under submergence and desubmergence stress, mitochondria signaling mutants *cdk2*/*rao1* and *anac017*/*rao2*, impaired in retrograde signaling to reprogram the nuclear transcription, have been shown to be very sensitive to hypoxia [50].

ANAC017 is activated by mitochondria perturbation and the transcriptional network regulated by ANAC017 responds to H_2_O_2_ cytosolic accumulation [51]. The activation of ANAC017 by endoproteolytic cleavage, for the migration from the endoplasmic reticulum into the nucleus, is likely mediated by mitochondrial generated ROS, through a mechanism that is still unknown [51].

A comparison between the transcriptome of the *cdk2*/*rao1* and *anac017*/*rao2* mutants and Arabidopsis accessions characterized by sensitivity to submergence, identified WRKY40 and WRKY45 among the commonly regulated genes [50]. The Arabidopsis mutants *wrky40KO*, *wrky45KO1* and *wrky45KO2* showed a high accumulation of H_2_O_2_ (measured with 3-3’-diaminobenzidine staining, DAB) under submerged and desubmerged conditions, together with a lower tolerance [50].

A further coordination between direct low O_2_ sensing and a ROS-dependent mechanisms require the hypoxia-responsive universal stress protein 1 (HRU1). HRU1 is a target of RAP2.12 and is involved in the regulation of ROS production under hypoxia [52]. Under aerobic conditions, HRU1 is localized in the cytosol as a homodimer. Under low O_2_ its transcription is enhanced, and HRU1 migrates as a monomer to the plasma membrane where it interacts with the NADPH oxidase protein respiratory burst oxidase homologue D (RBOHD) and Ras homologous (RHO)-like small G proteins of plants 2 (ROP2), which are both required for the production of ROS [53]. The lack of the HRU1 dimerization site in the *hru1-1* Arabidopsis mutant alters ROS production and increases the sensitivity of the *hru1-1* mutant plants to low O_2_.

Arabidopsis *rbohD* mutants are very intolerant to anoxia [46] and negatively affected in *ADH1* expression compared to wild type seedlings under waterlogging and hypoxia [41,54]. This suggests that, under these conditions, ROS produced by RBOHD may represent a positive signal required for plant tolerance to hypoxia.

The expression of a set of genes involved in oxidative stress response is induced in Arabidopsis plants subjected to flooding [55]. Interestingly, this set of genes is expressed at the seedlings stage and includes some that are target of RAP2.12. In adult plants, the expression of these genes is dampened by an unknown factor, likely as a result of a developmental-related stimulus. ERF-VIIs are thus positive regulators of the genes involved in the fermentative metabolism but also of oxidative stress-related genes, such as the zinc finger protein *ZAT12* and the glutathione S-transferase U24 *GSTU24*. However, this only happens in young plants. In this context, the age-dependent sensitivity of Arabidopsis to low O_2_ stress has been suggested to be dependent on the activity of ANAC017 [56]. Oxidative stress marker genes that are activated under submergence with the involvement of ANAC017 were shown to be located within heterochromatic regions in Arabidopsis submerged plants in the adult phase [56].

Three TFs belonging to the ERF-VII family, i.e., RAP2.2, RAP2.3 and RAP2.12, mediate the response to oxidative stress, where they likely act redundantly [57]. The overexpression of RAP-type ERF-VII confers tolerance to oxidative stress after H_2_O_2_ application [57]. It is interesting that oxidative stress was applied to five-day-old Arabidopsis plants, thus in the juvenile phase when genes related to ROS scavenging and signalling are positively regulated by ERF-VII [55].

Among the ERF-VII group, rice (*Oryza sativa*) SUB1A is remarkably not a target of the N-degron pathway [19]. SUB1A resistance to degradation is likely due to the C terminus interaction with the N terminus, which masks the region involved in the N-degron pathway [58]. However, *SUB1A*, which is up-regulated upon ethylene accumulation in submerged plants, plays a crucial role in enhancing rice tolerance to submergence. SUB1A controls carbohydrate consumption during the stress and dampens gibberellic acid (GA)-dependent stem elongation by enhancing the accumulation of GA signalling repressor slender rice 1 (*SLR1*) and SLR1-like *SLRL1* genes [59]. MPK3 interacts with, phosphorylates and activates SUB1A1, the allele involved in tolerance to submergence, upon submergence [60]. MPK3 together with MPK6 are known to be involved in ROS signalling in plants [61]. However, whether a MAPK kinase cascade, which is thought to be activated in the SUB1A1 pathway, is involved in a ROS-related response in rice under submergence has not been clarified.

Interestingly, the rice M202 line, harbouring SUB1A, shows a higher transcription of ROS scavenging enzymes (i.e., ascorbate peroxidases *APX1* and *APX2,* superoxide dismutase *SODA1*, and catalase *CATA* and *CATB*) when treated with methyl viologen (MV) that leads to the production of ROS in chloroplasts [62], suggesting at least a link between SUB1A and ROS detoxification.

## 3. ROS/NO Involvement in Adaptation to Environmental Hypoxia

**Figure 1:**
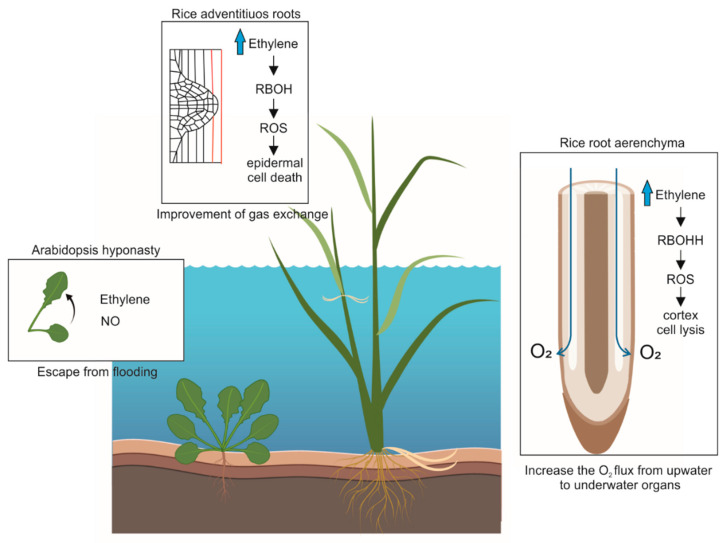
Role of reactive oxygen species (ROS) and nitric oxide (NO) in the environmental adaptation of plants to hypoxia: aerenchyma formation in rice [67], adventitious root emergence in rice [73], and leaf hyponasty in Arabidopsis [77]. The figure was created with BioRender.com.

During O_2_ shortage stress, e.g., total or partial plant submergence, waterlogging or flooding, some plants develop morphological and physiological adaptations in order to increase their capacity to produce ATP without O_2_ or to increase the supply of O_2_ to tissues to restore aerobic respiration. Anatomical adaptations are observed in several species: rice (*Oryza sativa*) develops additional aerenchyma [63], aimed at increasing the O_2_ flux to underwater organs; tomatoes (*Solanum lycopersicum*) shows a reduction in lateral roots and the development of adventitious roots instead [64] (Figure 1).

Aerenchyma formation is characterised by the creation of internal gas spaces that produce a path for O_2_ diffusion from above water to underwater organs [65]. Oxygen diffusion to submerged plant organs supports aerobic respiration in zones otherwise experiencing O_2_ shortage. In plants, aerenchyma of lysigenous origin results from programmed cell death. This differs from aerenchyma generated by schizogeny, which is the result of cell separation and the expansion of already existing air spaces. In rice, lysigenous aerenchyma is constitutive under aerobic conditions, but further induced under hypoxia. Lysigenous aerenchyma is regulated by ethylene and ROS in deep-water and lowland rice shoot tissues [66] and roots [67]. Rice varieties also vary in aerenchyma development regulation by ethylene and/or ROS. In particular, rice FR13A plants harbouring SUB1A1 appear to depend mainly on ROS activity for aerenchyma formation [68].

In rice roots under O_2_ deficiency, the NADPH oxidase RBOH isoform H (RBOHH) regulates the production of ROS involved in the subsequent formation of inducible lysigenous aerenchyma [67] (Figure 1). Under waterlogged conditions, plants produce ethylene, which accumulates due to slow gas diffusion in water [69], thereby stimulating the formation of lysigenous aerenchyma [63]. In addition, calcium (Ca^2+^) dependent protein kinases CDPK5 and CDPK13 work in synergy in cortical cells of roots in order to mediate the activity of RBOHH. The strong induction of ROS production, likely because of Ca^2+^ signalling activation, stimulates the formation of inducible aerenchyma under waterlogged conditions [67]. Aerenchyma formation through lysigeny is regulated by ROS in maize (*Zea mays*) roots under waterlogging [70]. In this condition, several genes related to ROS production and scavenging (e.g., *RBOH* and *MnSOD*) have been identified, suggesting that ROS play a role in waterlogging-related aerenchyma in maize as well. In addition, an induction in *RBOH* expression, with the parallel repression of the gene coding for a ROS-scavenging metallothionein, has been observed in maize roots, together with a reduction in aerenchyma after treatment with diphenyleneiodonium (DPI), an NADPH oxidase inhibitor [71]. Similar findings have been observed with wheat (*Triticum aestivum*) seedlings exposed to stagnant deoxygenated conditions [72]. In these conditions, ethylene and ROS signalling are involved in wheat acclimation to hypoxia resulting in the formation of lysigenous aerenchyma.

Under flooding, the formation of AR, which improves gas exchanges, has been observed in several plant species. In rice, AR emergence from the stem correlates with RBOH-produced ROS cell death, which is confined to the epidermal cells above the AR primordia (Figure 1). This likely facilitates subsequent root emergence, which involves the activation of a mechanical force [73]. In this mechanism, ethylene seems to play a role in promoting AR growth, but also in limiting cell death where AR emerges from the native organ [73].

HRE2, an ERF-VII TF, promotes AR elongation in Arabidopsis [74]. Overexpression of *HRE2* in air induces AR elongation, mimicking hypoxia, while ethylene inhibits this process. Hypoxia thus promotes AR elongation with the contribution of ERF-VII, while ethylene acts as an inhibitor to hypoxia-induced growth. Whether and how AR elongation interacts with AR emergence through ROS is an open question.

Many plants react to submergence by hyponastic growth, which includes the upward movement of leaves followed by petiole elongation, in order to escape from flooding and re-establish contact with air. In Arabidopsis, hyponastic growth has been shown to be mediated by ethylene [75,76]. Subsequently, an interaction among ethylene, NO and non-symbiotic haemoglobin GLB1/PGB1 has been found to influence Arabidopsis hyponasty under very low O_2_ [77]. NO emission rate was found to increase in Arabidopsis rosette under O_2_ level < 1%. At low O_2_ level, GLB1/PGB1 silencing lines Hg:Glb1 showed a higher emission rate of NO. In parallel, Hg:Glb1 plants showed a higher hyponastic response in the presence of ethylene. NO and ethylene thus modulate hyponastic growth in response to low O_2_ levels (Figure 1). Given that ethylene promotes the expression of *PGB1*, acting as a NO-scavenging enzyme [45], hyponastic growth triggered by ethylene is likely uncoupled from this mechanism. Hyponastic growth is therefore regulated by mechanisms that are both ethylene-dependent and independent, with the latter involving NO.

## 4. ROS/NO in Plant Development and in Hypoxic Niches Generated by Plant-Microbe Interactions

In plants, O_2_ shortage can be an endogenously generated physiological status that occurs chronically in organs or tissues during development. Shoot apical meristems (SAMs) [16] and meristems of lateral root primordia (LRP) [15] are characterised by chronic hypoxia where low O_2_ is continuous and probably under homeostatic control [17]. Hyperoxia treatment slows down the meristem activity of SAM and LRP, suggesting that hypoxia is a favourable state for these tissues [15,16].

SAMs require low O_2_ to produce new leaves through the activation of LITTLE ZIPPER 2 (ZPR2). ZPR2 is a target of the N-degron pathway and is thus stabilised by O_2_ shortage. Under hypoxia, ZPR2 interacts with class III homeodomain leucine zipper transcription factors (HD-ZIP III). HD-ZIP III target genes are involved in SAM activity and meristem size [16].

Interestingly, ROS play an important role in root apical meristems (RAM). At the RAM, ROS distribution is controlled by the root meristem growth factor 1 (RGF1)-inducible transcription factor 1 (RITF1). RITF1 gene expression modulates the redistribution of ROS throughout the developmental zone of the roots and therefore regulates, through oxidative post-translational modification, the stability of PLETHORA2, which is a key RAM regulator [78]. At the root meristematic zone, ROS are a key signal involved in establishing the size of the developmental zones, modulating the transition from proliferation to differentiation [79].

Recent results suggest that the precise accumulation and distribution of ROS is key for the maintenance of stem cell niche and the size of SAM [80,81], however knowledge of ROS regulation in the SAM is still limited [82].

Some interfaces between plants and microbes also represent hypoxic microenvironments. Arabidopsis crown gall tumors formed upon Agrobacterium tumefaciens infection show a steep drop level of O_2_, likely caused by high metabolic demand, which results in a hypoxic environment during gall formation and development [83]. A pentuple Arabidopsis erf-vii knockout mutant infected with Agrobacterium showed reduced symptoms. On the contrary, a significant increase in symptoms was observed in pco1pco2, prt6 and ate1ate2 mutants, suggesting that stabilisation of ERF-VII proteins contributes to gall development.

Similarly, the root tumor-inducing pathogen Plasmodiophora brassicae triggers a hypoxia-like response in Arabidopsis roots, with the induction of anaerobic genes during the infection [84]. In parallel, Arabidopsis erf-vii mutants infected with Plasmodiophora showed reduced symptoms, suggesting the involvement of ERF-VII in clubroot development. The role that ROS may play in relation to hypoxia in gall-forming pathosystems has not yet been investigated.

The symbiotic root nodule is an interesting example of the crucial importance of a balance between availability and protection from O_2_ [85]. The interaction between legumes and nitrogen (N_2_) fixing rhizobia in the plant roots leads to the development of the nodule structure, where bacterial enzyme nitrogenase reduces N_2_ to NH_3_, which is then assimilated by the plant [86]. This association is beneficial to plants, which offer rhizobia a carbon source and a microaerophilic environment. In fact, nitrogenase is sensitive to O_2_ and bacterial genes for nitrogenase assembly are only expressed at low O_2_ levels [87].

The low O_2_ nodule environment is maintained through an O_2_ diffusion physical barrier and the expression of symbiotic plant haemoglobin, which binds O_2_ [88]. NO has been detected in the functional nodules of several legumes, and its level increases under flooding [89,90,91]. Indeed, in alfalfa nodules, an alternative way of producing energy is the phytoglobin-NO respiration cycle, which functions partly under normoxia and fully under hypoxia [92]. What role ERF-VII plays in the nodule organ and how O_2_ and NO availability relates to this role is still unknown.

The plant immune system that responds to biotic cues operates through the recognition of extracellular molecular patterns and the activation of downstream responses, which include the production of ROS, likely acting both as antimicrobial agents and signals [93]. Botrytis cinerea, a necrotrophic fungal pathogen, is negatively affected by an early production of ROS. However, ROS can also lead to cell death, which is considered beneficial for necrotrophic fungi [94]. Remarkably, hypoxia is established at the site of *B. cinerea* infection [95], where the local nearly O_2_-free environment allows the stabilization of ERF-VII proteins. Although the pentuple erf-vii Arabidopsis mutant displays reduced tolerance to *B. cinerea*, enhanced stabilization of ERF-VII in 35S:ΔRAP2.12 plants does not enhance tolerance to *B. cinerea*. The activation of a hypoxic response may enhance the survival of the leaf tissue to hypoxia arising from pathogen infection or may be aimed at activating a still unknown plant defence pathway, requiring the activity of an O_2_-labile protein.

## 5. Post-Submergence ROS Production

The recovery phase from submergence, when the water recedes, is stressful for plants. During submergence, muddy water can impede photosynthesis, reducing the access of light to underwater organs. At post-submergence, the sudden availability of O_2_ and light can be challenging for survival [96]. A rapid burst of O_2_ and light impacts plant cells under recovery, which thus likely leads to the production of ROS. In parallel, the de-submergence phase can paradoxically lead to dehydration due to a drop in root hydraulic conductivity, even though there is still plenty of water available [96].

Knowledge of how plants respond to the post-submergence phase is still limited. Different accessions of Arabidopsis, namely Lp2-6 and Bay0, tolerate the post-submergence phase in different ways [97]. The respiratory burst oxidase RBOH isoform D is involved in the superior post-submergence recovery capacity of the Lp2-6 genotype compared to Bay0. In both plants, there is visible dehydration in older leaves, which are the most severely damaged organs in both genotypes. Intermediate leaves, which show the greatest difference between the two accessions, have been used to study the ribosome-associated transcripts. The results showed that differential ROS accumulation and antioxidant content (glutathione and ascorbate) are key to the recovery phase. There is a higher production of ROS in parallel with a high malondialdehyde (MDA) content (formed by ROS mediated degradation of polyunsaturated fatty acids) in the sensitive Arabidopsis accession [97]. RBOHD transcripts increase in the sensitive genotype, more than in the tolerant one in the recovery phase, which explains the excessive accumulation of ROS which is likely detrimental to tolerance.

However, when testing the rbohD mutant and the NADPH oxidase inhibitor DPI on the sensitive Arabidopsis genotype, it is clear that a limited and controlled ROS production after de-submergence is beneficial to survival. This again highlights the dual role of ROS, whose balance is key for adaptive and maladaptive responses.

In rice, desubmergence stress increases the abundance of ROS scavenging enzymes in SUB1A-harbouring genotypes, resulting in enhanced tolerance to oxidative stress [98]. Under reoxygenation, MDA and thus ROS-related damage appears to be higher in M202 rice plants than in the near-isogenic line M202(SUB1A). In parallel, the staining of O_2_- and H_2_O_2_ through nitroblue tetrazolium (NBT) and DAB, respectively, is higher in M202 plants. In contrast, the transcripts of antioxidants such as SOD, APX and CAT, are higher in M202(SUB1A) plants, suggesting that SUB1A is involved in oxidative stress tolerance under recovery from submergence to reduce harmful ROS accumulation.

## 6. Conclusions

ROS availability in cells is linked to O_2_ availability and as a consequence, their modulation, through production and scavenging, might be part of signalling under hypoxia. The availability of ROS (measured directly or indirectly) and NO, the variation in the antioxidant system, and the activation of the downstream signalling pathway have been detected under different regimes of O_2_ availability and water submergence and related to plants adaptation phenomenon (Table 1).

In the last few years, some important advances were made about the complex and multifaceted role of ROS/NO under low O_2_ that were highlighted in this review: (i) NO depletion is mediated by ethylene in order to pre-adapt Arabidopsis plants to hypoxia stress, through the enhanced stabilization of ERF-VII [45]; (ii) RBOHD is required for Arabidopsis tolerance to waterlogging, suggesting a crucial role for H_2_O_2_ accumulation [41]; (iii) ROS detoxification under flooding condition is under the indirect control of ERF-VII TF during the Arabidopsis plant juvenile phase [55]; (iv) mitochondrial respiration is a key cause of physiological Arabidopsis cell changes under hypoxia [36]; (v) a strong oxidative state of glutathione pool is observed in Arabidopsis leaves under hypoxia [36]; (vi) Arabidopsis ADH enzyme activity is under the control of redox modification [39]; (vii) Arabidopsis mitochondria retrograde signaling is involved in ANAC017 activation under low O_2_ and includes ROS signaling [50]; (viii) RBOHH dependent ROS production is crucial for rice lysigenous aerenchyma formation in low O_2_ conditions [67]; (ix) the balance between ROS production via RBOHD and scavengers is crucial for Arabidopsis recovery after submergence [97].

In parallel, some very recent results may imply a role for ROS/NO that has not yet been identified: (i) Arabidopsis lateral root primordia are characterized and regulated by a chronic hypoxic state [15]; (ii) Arabidopsis shoot meristem requires hypoxia to regulate the production of new leaves [16]; (iii) Arabidopsis HRE2 ERF-VII TF promotes adventitious roots elongation under hypoxia [74]; (iv) Arabidopsis ERF-VII are involved in galls formation, which is characterized by a hypoxic condition [83,84]; (v) Botrytis cinerea necrotrophic pathogen induces local hypoxia in Arabidopsis leaves [95].

The main challenge is at present to understand whether the availability of ROS and NO directly influences the system of direct O_2_ sensing in plants, guided by the PCO/ERF-VII coordination, and if ROS represent an additional sensing mechanism driving other genes than the hypoxic core.

Evidence that ROS might act through a signalling mechanism in parallel to direct O_2_ sensing is the fact that morphological adaptations to hypoxia, such as aerenchyma, are based on the presence of ROS, but are currently found to be independent of PCO/ERF-VII. In these cases, the location and timing of ROS are designed to exert cell death without resulting in uncontrolled reactions. Moreover, the possibility that redox-based modification can influence the activity of proteins downstream ERF-VII transcriptional regulation has emerged.

However, in mammalian cells, ROS likely have a role in modulating the O_2_ sensing mediated by HIFs. In plant cells, this is true for NO, which, together with the O_2_ level, is involved in regulating the stability of ERF-VII. This possibility is still to be evaluated for ROS.

The role of hypoxia in defining plant development represents an exciting topic of research. In these microenvironments, O_2_ homeostasis drives the developmental phases. Defining the role of ROS and NO in hypoxic niches represents an opportunity for future investigations.

## Figures and Tables

**Table 1:** Reactive oxygen species (ROS) and nitric oxide (NO) involvement in low oxygen (O_2_) Arabidopsis and rice adaptation phenomenon.

Low O_2_ Related Aspect	ROS/NO-Related Aspects	Phenomenon	References
Proteolytic control of ERF-VII	NO availability	ERF-VII degradation in Arabidopsis	[10]
Hypoxia response priming	NO depletion mediated by ethylene	ERF-VII stabilisation in Arabidopsis	[45]
Mitochondria-triggered hypoxia signalling	ROS production and MPK6 activation	Arabidopsis seedlings survival	[47]
Mitochondria-triggered hypoxia signalling	ROS production and ANAC017 activation	Arabidopsis tolerance at the juvenile stage	[50,56]
RBOH-triggered hypoxia signaling	ROS production	Arabidosis hypoxia tolerance	[41,46,53,54]
Anoxia signalling	ROS production	HSFs, HSP-mediated protection in Arabidopsis	[48]
Environmental hypoxia	ROS production through RBOH	Inducible lysigenous aerenchyma formation in rice	[67]
Environmental hypoxia	ROS production through RBOH	Adventitious roots emergence in rice	[73]
Environmental hypoxia	NO availability	Hyponastic growth in Arabidopsis	[77]
De-submergence	ROS detoxification	Survival in rice and Arabidopsis tolerant plants	[97,98]
